# Rediscovery of two casts of the historically important ‘*Proteo-saurus*’, the first complete ichthyosaur skeleton

**DOI:** 10.1098/rsos.220966

**Published:** 2022-11-02

**Authors:** Dean R. Lomax, Judy A. Massare

**Affiliations:** ^1^ Department of Earth and Environmental Sciences, The University of Manchester, Oxford Road, Manchester M13 9PL, UK; ^2^ Earth Sciences Department, State University of NY, College at Brockport, NY 14420, USA

**Keywords:** ichthyosaur, *Proteosaurus*, *Ichthyosaurus*, Early Jurassic, Mary Anning, Everard Home

## Abstract

The first complete ichthyosaur skeleton was introduced to the scientific community in 1819 by Sir Everard Home, and given the name *Proteosaurus*, although the name was subsequently replaced by ‘*Ichthyosaurus*’. The skeleton is from Lyme Regis and was probably collected by Mary Anning as it was in the collection of Colonel Birch. The specimen ultimately ended up in the collection of the Royal College of Surgeons, London, where it was destroyed in a bombing raid during World War II. We have discovered two plaster casts of the specimen, although no record exists of casts ever being made. The casts are at the Peabody Museum, Yale University, USA and the Museum für Naturkunde, Berlin, Germany. Significantly, these verify the accuracy of the published drawing of the specimen, and clarify morphologies of some of the bones. Discrepancies between the drawing and the casts are mainly in the details of the forefins and hindfins. The specimen can be assigned to *Ichthyosaurus*, but the species cannot be determined. This case illustrates the importance of old casts in museum collections. Additional, yet unrecognized casts of this specimen might exist in the UK or elsewhere.

## 1. Introduction

Fossils that can now be identified as ichthyosaurian were illustrated as early as 1699, and more material was collected throughout the eighteenth century in southern Germany and the UK, although their affinities were not understood [1–4]. Ichthyosaur fossils from southwestern England, particularly from the Lyme Regis–Charmouth area of the Dorset coastline, played an important role in the development of an understanding of prehistoric life and the establishment of palaeontology as a scientific discipline. Ichthyosaurs, with their clearly reptilian skull but a fish-like body, were enigmatic creatures that caught the attention of many amateur naturalists, collectors and scientists of the nineteenth century. In the UK, their popularity with the public initially exceeded that of dinosaurs, given the abundance of newly found fossils, with the first ichthyosaurs described more than 20 years before the word dinosaur was invented.

The earliest scientific accounts of ichthyosaurs were papers written by Sir Everard Home, a British surgeon, who presented his findings in a series of papers that were read at meetings of the Royal Society of London [5–9]. These and other early reports shifted thinking towards a scientific approach to the study of fossils. Home was probably influenced by his brother-in-law John Hunter (1729–1793), a surgeon and anatomist, whose osteological and palaeontological collection (which included marine reptile fossils) became the Hunterian Museum of the Royal College of Surgeons, London [1–4]. Notably, Home [5] provided the first scientific study, description and illustration of an ichthyosaur: a large skull and some postcranial material collected by Joseph and Mary Anning from Lyme Regis, Dorset in 1811 and 1812 [1,10], now the holotype of *Temnodontosaurus platyodon*. Home initially thought it was a crocodile [5], then some type of fish, and later concluded that it was a link between fishes and crocodiles.

In 1819, Home presented two additional papers (4 March, 1 April) that discussed three other ichthyosaur specimens. These specimens were briefly described in the first paper [7] and they were illustrated and their affinities were discussed in the second [8]. One of the specimens described was a nearly complete skeleton that is the subject of this report. The specimen was the first skeleton of an extinct marine reptile in the scientific literature and the most complete ichthyosaur skeleton known at the time. Unfortunately, this historically significant specimen was almost certainly destroyed during World War II [11,12]. However, we have located two casts of the specimen in museum collections, the significance of which had not been recognized. Here, we discuss the history of the skeleton, identify and describe the casts and evaluate the taxonomy of the specimen.

## 2. History of the first complete ichthyosaur skeleton

Home's [5] initial presentation resulted in him being asked to study additional specimens, and these became the subject of his subsequent papers. Home himself [7] described how he acquired specimens to examine:

‘In the year 1814, the skull and vertebrae of this fossil skeleton were first described in the Philosophical Transactions; and so much was the attention of the public called to the subject by that account, and so many specimens were brought under my observation, that in the year 1816, I was enabled to make many valuable additions to my former paper. In 1818, I laid before the Society the description of bones not before met with; and since that time, through the kindness of Mr. De la Beche, and Colonel Birch, I have procured materials, which put it in my power to describe nearly the complete skeleton, and to correct any errors, which the imperfect state of the first specimens had led me to commit.’ [7, p. 209].

With the additional specimens, Home concluded that these animals formed a link between lizards and salamanders. He proposed the name ‘*Proteo-saurus*’ for these specimens and for this name to be used for other ichthyosaurs known at the time [7,8]. The name derives from the Proteidae, a family of salamanders whose vertebrae Home thought were similar to those of ichthyosaurs, so much so that he figured proteid vertebrae on the same plate ([8], plate XV, figs. 2,3) as the ichthyosaur specimen that is the focus of this study. His conclusion that ichthyosaurs were a link between reptiles and amphibians differed from prevailing thought at the time [1,4,13]. However, in the previous year, König [14] had proposed the name *Ichthyosaurus*. Even though ‘*Ichthyosaurus*’ was initially a nomen nudum [2], and thus not valid by modern standards, that was the name used by subsequent workers (e.g. [15,16]) and is the name that was retained. As a consequence, *Proteosaurus* is considered a nomen oblitum [2] and has never been used since Home's pioneering work.

Home's early attempt to understand the affinities of ichthyosaurs was aided greatly by the first complete specimen, mentioned in the previous quote, and which is the focus of this paper:

‘A specimen belonging to Colonel Birch, which in compliance with the wishes of my friend Mr. De la Beche has been brought under my observation, contains nearly the entire skeleton of this extraordinary animal, and shows the important fact, that it had posterior as well as anterior feet; as it gives a posterior view, the bones forming the pelvis cannot be made out, but these may be said to be the only ones with which we are now not acquainted.’ [7, pp. 210–211].

This brief description emphasized the significance of the specimen being the most complete, but also the first to show all of the bones in place, including the hindfins, which were previously unknown. It also records that at the time, the specimen belonged to Lt-Col. Thomas James Birch. Birch had amassed a large collection of fossils from Lyme Regis, Dorset, which he had primarily acquired from Mary Anning and her family [10,12]. This included the specimen figured by Home, which Birch acquired in 1818 ([4, p. 39], [10–12]). In September of that year, De la Beche transported the specimen to London for Home to study [17]. Although no stratigraphic information exists for this specimen, historic specimens from Lyme Regis are assumed to be from the upper Hettangian to lower Sinemurian stages of the Lower Jurassic [18]. However, strata from the lower Hettangian to Pliensbachian (Lower Jurassic) are also exposed along the coast in the Lyme Regis area [19].

In 1819, Birch visited the Anning's fossil shop and found them selling many of their possessions, including furniture, to pay their rent. As a result, Birch decided to sell his fossil collection and donate the proceeds to the Annings [10,12]. An auction was held on Monday 15 May 1820 at Bullock's Egyptian Hall in Piccadilly, London [12]. The ichthyosaur skeleton of interest was part of the auction, listed as lot number 102, with a brief description:

‘This skeleton presents a most interesting illustration of the osteology of the Icthio-saurus, or Proteo-saurus; it was the subject of a celebrated Paper addressed to the Royal Society, by their Vice President, Sir Everard Home, Bart. & and a very fine engraving of it, from a drawing by Mr. Cliff, is published in the Philosophical Transactions for 1819.’ [20].

The ichthyosaur skeleton did not sell at the auction because it had not reached the minimum price Birch had placed on it [12]. Instead, it was purchased after the auction for the Museum of the Royal College of Surgeons, London [10]. A record of the acquisition exists in Mr William Clift's diary at the Royal College of Surgeons, which states that the specimen entered the collection on Friday 9 June 1820: ‘*Received from Mr Bullock- Col Birch's fossil of Proteo-saurus (lot 102) £100.0.0*’, although different prices have been reported [17]. Very little has been published on this specimen following the original paper by Home [8], though it was listed as specimen number 156 in Owen's catalogue [21, p. 42]. Interestingly, the specimen is also identified in the catalogue as a young *Ichthyosaurus intermedius*, a species that is now synonymized with *I. communis* [18,22]. The original illustration in [8, plate XV] was also reproduced in Howe *et al*. [1, p. 16] and Evans [3, fig. 9].

Unfortunately, the specimen was subsequently destroyed in an air raid in May 1941 during World War II [1,11,12]. There is no written evidence to suggest that casts of the specimen were ever created. This is unusual considering the importance of the specimen and that various casts were created for many other ichthyosaur and plesiosaur specimens collected during the early nineteenth century (e.g. [23,24]).

## 3. Description of specimen

**Figure 1.**
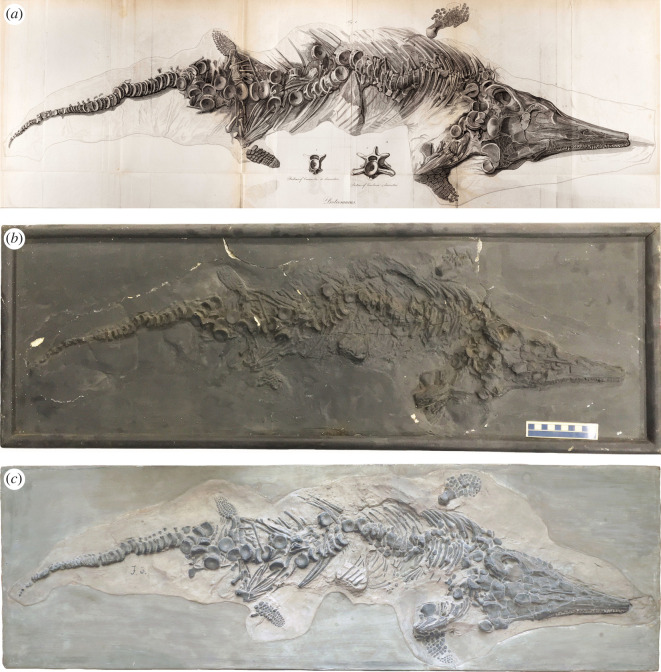
(*a*) Original William Clift drawing of the first complete ichthyosaur skeleton discovered ([8], plate XV, fig. 1). Image reproduced from Home, E (1819) Reasons for giving the name *Proteo-Saurus* to the fossil skeleton which has been described. *Phil. Trans. R. Soc.* **109** 212–216. (*b*) YPM VP.001989, the Yale cast of the specimen. (*c*) MB.R.1891, the Berlin cast of the specimen. All figures at the same scale: 10 cm scale bar shown in (*b*).

The specimen was figured by Home ([8], plate XV, fig. 1), who further noted in the plate description that it was ‘*more entire than any hitherto met with*’. With that said, Home did not provide a more thorough description of the specimen in print, and explained why in the plate caption:

‘The different bones of which it is composed are sufficiently perfect, and sufficiently in their places, to make any verbal explanation unnecessary.’ [8, plate XV caption].

However, we feel it is necessary to provide a brief description of the skeleton based on the ‘*natural size*’ illustration drawn by William Clift (as credited in the plate XV caption in [8]) and engraved by J. Basire. The practically complete skeleton is exposed largely in dorsal view (figure 1*a*). The entire vertebral column is associated, but portions are displaced so that several cervical, posterior dorsal and anterior caudal vertebrae are lying flat in the matrix. The caudal series is almost completely articulated to the end of the tail. Ribs are preserved on both sides of the column. The skull is dorsoventrally flattened with numerous teeth preserved in the jaws. The rostrum is broken anteriorly. On the illustration, a shaded outline at the tip of the rostrum suggests that this portion was missing. Both forefins are present, with the right (lower in figure 1*a*) being the most complete of the two. Similarly, both hindfins are nearly complete. The left humerus and right femur appear well exposed and clearly show their respective morphologies.

Interestingly, Home [7] had stated that the only missing portion of this skeleton was the pelvis (see full quote above). However, the specimen also lacks all of the bones of the pectoral girdle, although because the specimen is preserved in dorsal view, they might have been buried under it. Moreover, two bones from the pelvis can be seen in the illustration: the left ilium covers the shaft of the left femur and the right ischium is lying under ribs. Given that this was the first complete skeleton of an ichthyosaur, it is plausible that Home had overlooked these bones that are mixed in with smaller ribs. A year later, studying additional material, he did identify the pelvis of an ichthyosaur, although he did not provide a description [9, p. 163].

Because the specimen was preserved in dorsal view, there is no evidence of a downward bend in the vertebral column that supported a tail fluke. This must have influenced interpretations by subsequent researchers. For example, Owen [25] examined several specimens that showed a distinct downward bend in the vertebral column, but concluded that the bend was a post-mortem effect, caused by the ligaments of a fin attached to a straight vertebral column. In fact, nineteenth-century reconstructions of ichthyosaurs showed a straight tail [26]. This interpretation persisted until the late 1800s, when skin impressions of a heterocercal caudal fin were found on specimens from southern Germany [27, pp. 194–195].

## 4. The casts

As part of our research on Early Jurassic ichthyosaurs, we have visited most museums and universities in the UK that have palaeontology/geology collections, but have not come across any casts of this ichthyosaur. Despite representing an important British specimen, the two surviving casts were found outside of the UK. One discovered by us in 2016 in the collections of the Peabody Museum, Yale University (YPM VP.001989), New Haven, CT, USA, and the other discovered by DRL in 2019 at the Museum für Naturkunde (MB.R.1891), Berlin, Germany. For simplicity, we refer to them as the ‘Yale cast’ and ‘Berlin cast’ in the discussion below.

Based on comparison of the two casts with the original illustration, it is clear that they are replicas of the *Proteosaurus* specimen discussed and figured by Home ([8], plate XV, fig. 1). However, the condition of the casts differs substantially (compare images in figure 1). Considering that the original was destroyed during World War II, it is somewhat ironic that the cast in the best condition is in the Berlin Museum.

### 4.1. The Yale cast

The specimen is recorded as *Ichthyosaurus* sp. from the Lower Lias of Lyme Regis, England in the YPM Ledger 1 (1908–1968). However, there is no mention of YPM VP.001989 being a cast (D. L. Brinkman, DRL 2017, personal communication), although it can be confirmed as a cast by the smooth surface and lack of detail, as well as by several cracks that expose the plaster.

According to archival material provided by museum assistant Dan Brinkman, the cast was part of a very large collection (over 90,000 specimens, the vast majority of which were invertebrate fossils) purchased by Prof. Charles Schuchert from the estate of his long-time acquaintance, Frederick Braun, a private collector and dealer from Brooklyn, NY, USA. The entire collection was donated to YPM in 1930, by Prof. Schuchert, and now comprises the ‘Schuchert-Braun Collection’. The vertebrate palaeontology division acquired 278 specimens from the Braun collection, one of which was YPM VP.001989. However, the list of specimens also included fossils from various historic localities in the UK that were often traded on the commercial fossil market. This suggests that the cast was originally made in the UK and was shipped to the USA. In fact, the YPM accession record (YPM 04 207) indicates that at least a portion of Braun's collection was purchased from E. B. Hall, a collector from Wellsville, NY, so either one of them could have purchased the cast from another collector or museum. No further details about the cast are known.

The cast is somewhat worn, bearing numerous cracks as well as some minor damage to the ‘bones’, exposing the white plaster beneath the uniformly dark grey colour of the cast (figure 1*b*). The cast has been embedded into plaster and mounted in a wooden frame measuring an outer length of 100.5 cm (inner length 96.4 cm) and outer width of 34.8 cm (inner width 30.5 cm). There is an outline distinguishing the original cast from the surrounding plaster, made more visible through the discoloration of the plaster, and it follows the outline of the specimen on the illustration in [8] (plate XV, fig. 1; figure 1*a,b*), except that the cast shows matrix extending around the left (upper) forefin.

**Figure 2.**
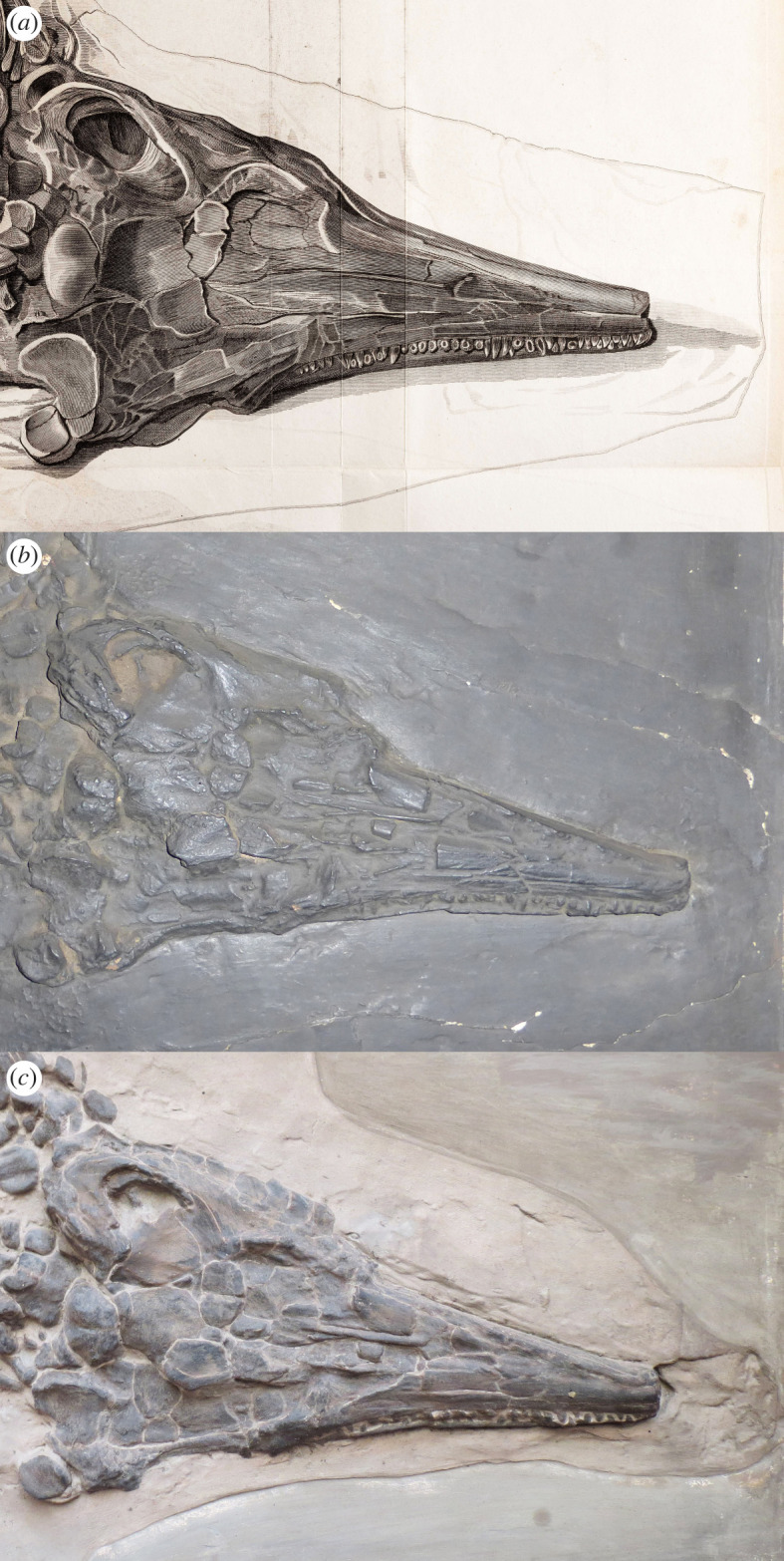
Comparison of skulls. (*a*) Skull in the original illustration ([8], plate XV, fig. 1). (*b*) Skull on YPM VP.001989, the Yale cast. (*c*) Skull on MB.R.1891, the Berlin cast.

**Figure 3.**
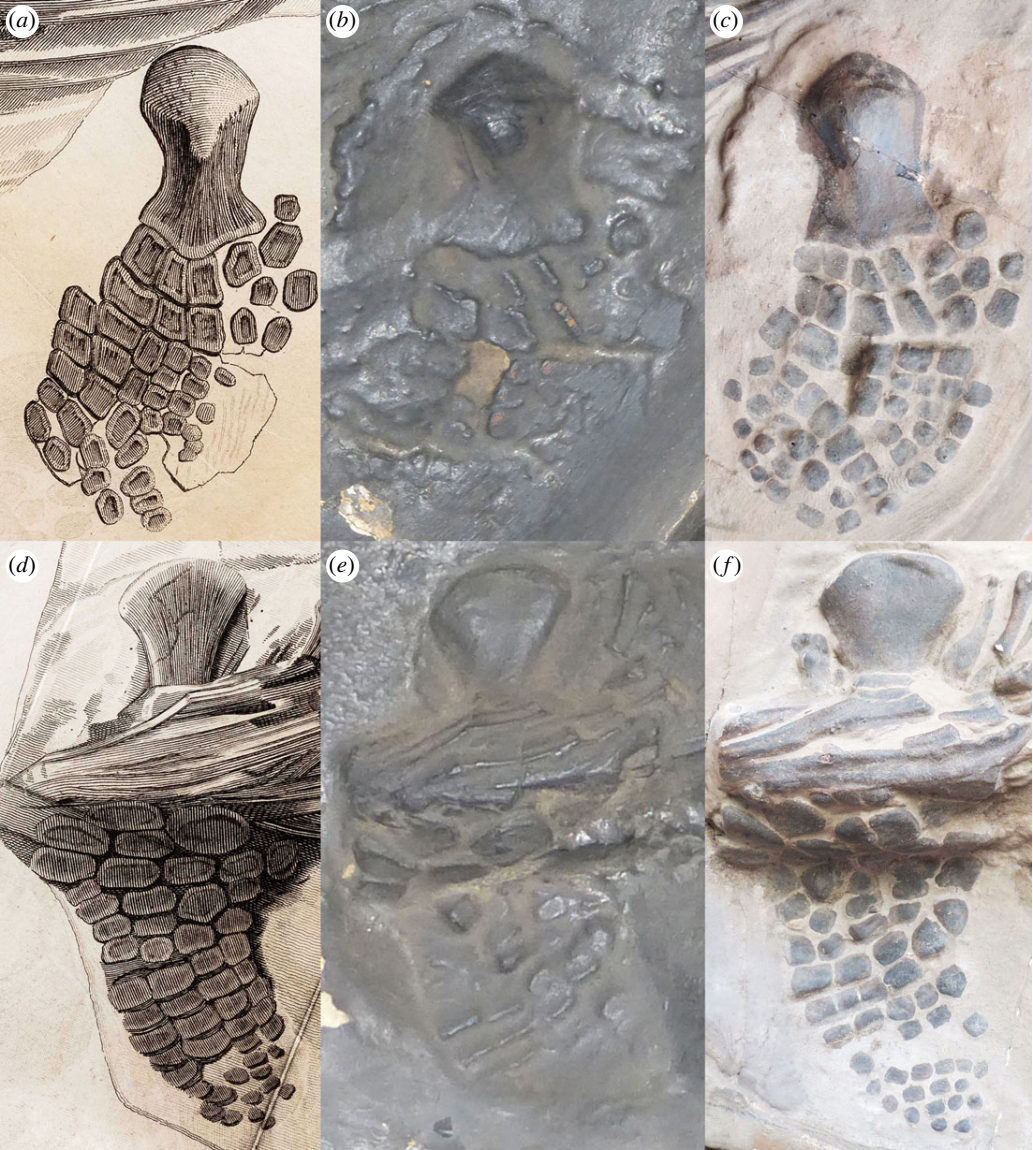
Comparisons of the forefins. (*a*) Left forefin in the original illustration (8], plate XV, fig. 1). (*b*) Left forefin on YPM VP.001989, the Yale cast. (*c*) Left forefin on MB.R.1891, the Berlin cast. (*d*) Right forefin in the original illustration. (*e*) Right forefin on the Yale cast. (*f*) Right forefin on the Berlin cast.

**Figure 4.**
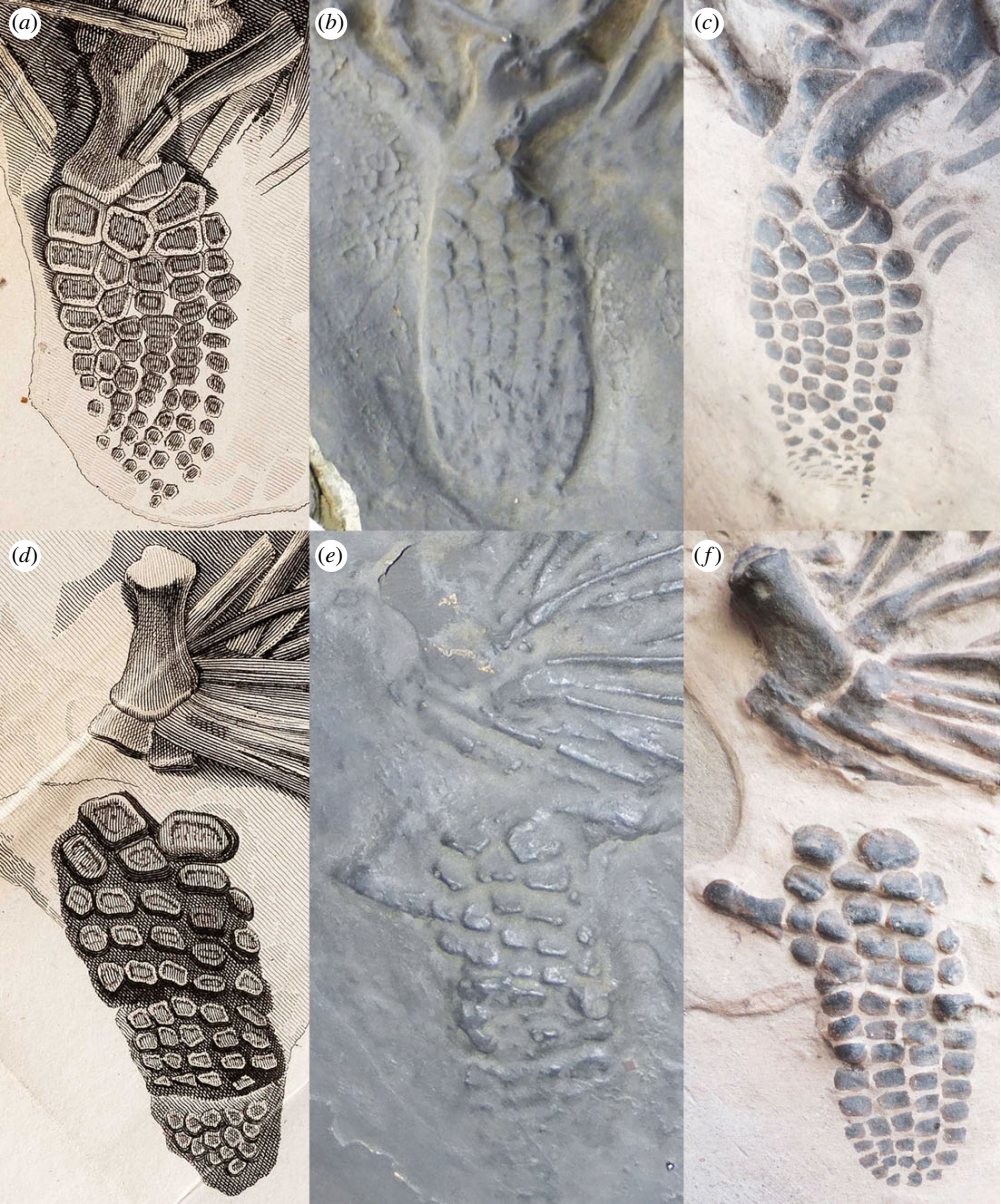
Comparisons of the hindfins. (*a*) Left hindfin in the original illustration ([8], plate XV, fig. 1). (*b*) Left hindfin on YPM VP.001989, the Yale cast. (*c*) Left hindfin on MB.R.1891, the Berlin cast. (*d*) Right hindfin in the original illustration. (*e*) Right hindfin on the Yale cast. (*f*) Right hindfin on the Berlin cast.

Due to the condition of the cast, only a few measurements of the skeleton could be taken (table 1). Compared with the original illustration ([8], Plate XV, fig. 1; figure 1*a*), the skeleton is preserved in exactly the same position, although some parts of the cast show more detail than others. For example, the dorsoventral crushing to the skull seen in the original illustration can be observed in the cast and some individual bones, such as the basioccipital, can be identified. However, the teeth are poorly defined and show little or no detail, unlike that seen on the illustration (figure 2*a,b*). The cast lacks an impression in the matrix defining a portion of the rostrum that is missing, as is shown in the illustration. Similarly, although both forefins are present, it is difficult to see much of their morphology or full extent, especially the smaller, more distal phalanges shown on the illustration. The right forefin (lower) is better preserved than the left, although the humerus is better exposed on the left (upper) forefin (figure 3*b,e*). Both femora are poorly defined. On the left hindfin (upper) the distal elements are indistinct and poorly defined, whereas on the right hindfin, they are only hinted at by irregularities on the surface of the cast (figure 4*b,e*). Overall, most of the skeleton appears smooth and lacks much of the finer details of the bones seen in the original illustration, which suggests that YPM VP.001989 is either a cast of a cast or that it is a very early cast made directly from the original early in its history.

**Table 1.** Select measurements (in cm) of known casts of the ‘*Proteo-saurus’* specimen figured by Home ([8], plate XV, fig. 1). Minor differences in measurements are probably due to differences in the quality of the casts.

	Yale cast YPM VP.001989	Berlin cast MB.R.1891
total length (tip of snout to tip of tail)	90.5	89.5
total length (along vertebral column)		94.5
skull length (preserved)	21.0	21.0
preorbital length (preserved)	12.0	
jaw length (preserved)		21.0 L /20.5 R
left humerus length	2.9	2.9
proximal width	1.7	1.8
distal width	1.9	1.9
shaft width	1.3	1.4
right forefin entire length		9.5
right femur length		2.0
proximal width		1.0
distal width		1.3
shaft width		0.9
left hindfin length (excluding femur)	4.7	6.1
ischium length		3.0

### 4.2. The Berlin cast

The only information associated with MB.R.1891 is that it is a ‘plaster cast of an ichthyosaur skeleton from an unknown location’; it also has ‘J. 3’ painted on the cast. Unfortunately, there is no record of the history of MB.R.1891 in the museum collection, in either the old entry catalogues or any other museum records (D. Schwarz, DRL 2021, personal communication).

The cast is in excellent condition, exhibiting no damage or deterioration. As in the Yale cast, a clear outline surrounds the original cast, which has been embedded in plaster in the shape of a rectangle. Given the overall shape, it is possible that the cast might have originally been set into a wooden frame, similar to the Yale cast. The block is 97 cm long, 30.5 cm wide and 2.5 cm deep, which compares favourably to the inner length (96.4 cm) and width (30.5 cm) of the frame on the Yale cast. The tan coloration of the matrix, as well as the light grey plaster surrounding it, contrasts with the dark grey to black colour of the bones and is similar to the contrast on the original illustration (figure 1*a,c*). A grey or dark grey matrix is more typical of Lyme Regis specimens.

The excellent condition of the entire cast allows for some key measurements and observations to be made (table 1). The skull morphology matches well with the original illustration, but it lacks the impression of the missing tip of the snout. Some bones can be clearly identified, such as the basioccipital and the area around the left temporal region (figure 2*c*). Due to the crushing and orientation of the skull, not much can be said about the skull taxonomically; the broken elements make it impossible to identify many of the individual skull bones. However, both forefins are preserved and provide taxonomically useful information (figure 3). They each have at least five digits, although the proximal region of the right forefin (lower) is partly buried by ribs, as in the original illustration (figure 3*d,f*). The left forefin (upper) is odd in that it shows five or more elements in contact with the humerus, a morphology unknown for any ichthyosaur (figure 3*b*). This could be because accessory digits were displaced or because additional bones were painted onto the cast. The left humerus is well preserved with a prominent dorsal process and a distal end that is only slightly wider than the proximal end (figure 3*c*).

Both hindfins appear well preserved and fairly complete, matching more closely with the morphology and extent seen in the original illustration than that of the Yale cast (figure 4). However, some of this completeness is due to interpretations made during the painting of the cast (see below). The right hindfin (lower) has at least four digits, including a bifurcation of the anterior digit (figure 4*f*). The left hindfin (upper) is almost identical to the illustration except for the femur and shows at least six digits, including at least two bifurcations (figure 4*a,c*). A pelvic bone, probably the ischium is dorsal to the right femur, below a rib. It is longer than the femur (table 1), bulbous proximally, narrow along the shaft and slightly flared distally (figure 1*c*).

The Berlin cast probably represents a later cast of the specimen, made using newer methods that better captured the relief and details of the specimen. As an example, the right femur on the Berlin cast shows more relief and better definition than that of the Yale cast (compare figure 4*e,f*). Some of the apparent definition, however, is because of the painting. It is likely that the artist used the original plate as a guide. Some of the distal phalanges on the forefins and hindfins, for example, are painted onto the cast where there is no obvious relief to indicate a bone was present (figures 3*c,f*, 4*c,f*). Similarly, to the left and adjacent to the right hindfin, there is a bone that shows more relief on the Berlin cast than on the Yale cast (compare figure 4*e,f*). A careful look, however, reveals that the bone is only partially painted on the Berlin cast. Just like Clift's original drawing in [8], the Berlin cast reflects a lot of interpretation by the artist.

### 4.3. Discrepancies between the original illustration and the casts

In Home's [8] plate caption, there was never any mention that the original specimen had been mounted in a wooden frame, a practice which was popular in the nineteenth century and is common with historic specimens. Clearly, the Yale cast must have been recognized as important to have a purpose-built wooden frame created for it. Although it is difficult to verify the full extent of the matrix outline of the Yale cast, it appears to follow the same outline as on Home's illustration, except for some additional matrix extending around the left forefin. The outline in the Berlin cast is much more defined. However, the outline in the Berlin cast shows more ‘matrix’ surrounding the specimen, especially dorsal to the skull and anterior vertebral column, and enclosing the left forefin (upper) and the right hindfin (lower) (figure 1*a,c*). Interestingly, the impression in the matrix of the missing portion of the rostrum shown in the illustration does not occur on either of the casts. Thus, the inferred length of the rostrum in the illustration is speculation.

In both casts, the left forefin (upper) is lying in matrix that is slightly raised above the level of the matrix surrounding the rest of the specimen, and a distinct outline surrounds it in the embedded plaster (figure 3*b,c*). This is of interest because, in the original illustration, the left forefin is placed outside of the matrix outline, ‘floating’ adjacent to the ribs with no indication of the raised ridge of matrix seen in both casts (figure 1*a*). It could be argued that the original forefin might have become detached and was simply reset next to the skeleton, or that a forefin from another specimen was added to make this specimen more complete. Numerous composites from historic specimens have been documented in museum collections (e.g. [28–31]) and some nineteenth-century collectors commonly added additional parts to a skeleton to make it appear more complete [28,32]. On both of the casts, however, the forefin is surrounded by matrix that is an extension of the matrix surrounding the skeleton (figure 1*b,c*), indicating that the forefin belongs with the rest of the specimen.

There are other notable inconsistencies between the original illustration and the casts. Although the vertebral column is generally similar in the casts and illustration, a few vertebrae near the hindfins are lying flat on the matrix in the illustration, whereas on the casts, they are slightly rotated into the matrix. The end of the tail has a couple of additional centra on the Berlin cast compared with the Yale cast, but the illustration has many more tail centra than on either cast (figure 1).

In the illustration, the left humerus is much longer relative to distal width, has a narrower shaft on the humerus and has a more bulbous head than on either cast. The facets for the radius and ulna are also at a more acute angle, resulting in a completely different morphology (figure 3*a–c*). On the right forefin of the Berlin cast, five digits are present with spacing between most of the elements, whereas the same fin in the illustration shows only four digits (except for an isolated phalanx positioned slightly under the ribs), closer spacing and fewer elements (compare figure 3*d–f*). Only the proximal fin elements and a few more distal disarticulated ones can be discerned on the Yale cast. Many seem to be rotated into the matrix. The left forefin (upper) of the Berlin cast shows as many as five elements in contact with the humerus, whereas the original illustration shows four elements in contact (figure 3*a,c*); neither scenario is known for any ichthyosaur. The left forefin on the Berlin cast is also wider, with more digits, than in the original illustration (figure 3*a–c*). By contrast, the left forefin on the Yale cast shows two fin elements adjacent to the humerus, lying on end in the matrix, but the sizes cannot be determined because only the ‘depth’ dimension is shown (figure 3*b*). At least two small round elements, probably from an accessory digit, are slightly posterior to the humerus. The second row of three elements are similarly rotated (figure 3*b*). This configuration is the common morphology in ichthyosaurs. Thus, the left forefin on the illustration was misinterpreted and the Berlin cast was painted to reflect what was in the illustration.

On the Yale cast, the right femur is poorly defined and shows no morphology, quite different from the Berlin cast (figure 4*e,f*), and probably is a result of the casting process. Significantly, the right femur is more slender, more symmetric, and better defined in the illustration than on the Berlin cast, resulting in two distinctly different morphologies and pointing to an inaccuracy in the original illustration (compare figure 4*d–f*). The illustration also appears to show an epipodial in contact with the femur, which we identify as a rib on both casts, based on its elongate shape (figure 4*d–f*). The left femur is covered by what looks like an ilium on the original drawing. The left femur is poorly defined on the Yale cast, but the shaft appears to be covered by a long, indistinct bone. On the Berlin cast, these bones are not painted correctly. Two bones seem to articulate with the elements of the fin, an impossible morphology. Actually one bone, the ilium, is overlying another, the femur (compare figure 4*a–c*). The remaining portion of the hindfins of the Berlin cast match fairly closely with the illustration, although there are some minor differences. The right hindfin (lower) shows a couple of additional elements compared with the illustration, and there are more ‘distal phalanges’ in the left hindfin (upper). As mentioned above, these extra elements have been painted onto the plaster (figure 4*c,f*). The proximal portion of the right (lower) hindfin on the Yale cast is similar to the illustration, although the bones are indistinct. The distal portion is little more than irregularities on the surface of the cast, unlike the illustration (figure 4*d,e*). The proximal half of the left (upper) hindfin is similar to the illustration, but, even though the distal elements are poorly defined, the fin does not have the same tapered shape shown in the illustration (figure 4*a,b*). On the illustration, there is also an oddly shaped bone proximal to the left femur that has a morphology similar to a femur. However, on the Berlin cast this appears more likely to be two neural spines, which are associated with two vertebrae (figure 1*a,c*).

Other minor discrepancies that we could identify with confidence were (i) a small, isolated bone (probably a fragment of rib) lying posterior to the right (lower) hindfin, which is not shown in the original illustration but is found in both casts (compare figure 4*d–f*); (ii) three extra rib fragments and a wider bone posterior to the left hindfin on the Berlin cast, but are not on the illustration nor apparent on the Yale cast (figure 4*a–c*); and (iii) although present on the Berlin cast, several of the ribs have not been painted to match the original illustration.

## 5. Discussion

Important, historic ichthyosaur specimens have been overlooked, even in major collections. For example, we found the missing holotype of *Suevoleviathan integer* on display at the Museum of Comparative Zoology, Harvard University, misidentified as *Stenopterygius* [33]. Similarly, an *Ichthyosaurus breviceps*, sold by Mary Anning and figured in William Buckland's famous Bridgewater Treatises [34], was not noticed in the collections of the Natural History Museum, London [35]. It did not even have an accession number until McGowan's work in the early 1970s. Another Anning specimen of *I. breviceps*, at the Sedgwick Museum, University of Cambridge, was mentioned and figured by Price [36] but did not have an accession number for over 150 years until we formally identified and described the specimen [37]. We also discovered a cast of the now destroyed holotype of *Ichthyosaurus latimanus* in the Derby Museum and Art Gallery [24]. It is not really surprising, though, that old casts, sometimes with deteriorating plaster, are not given much attention in museum collections.

Sending casts of important specimens to various museums was commonly done in the nineteenth century, both to disseminate information on important fossil discoveries and to provide display specimens at a time when museums were expanding [23,38,39]. Old casts in collections can be significant, especially if the original specimen has been lost or destroyed. This was the case for the specimen described herein, as well as for specimens similarly destroyed at the Bristol Museum during World War II (e.g. [23,24,40]). Casts of a skull and forelimb of ‘*Plesiosaurus’ megacephalus* (now *Atychodracon megacephalus*) along with a photograph of the entire specimen are the only record of a plesiosaur destroyed in the bombing of the Bristol Museum during World War II. The cast provided enough detailed morphological information that a new genus could be erected [40]. Taylor and Evans [41] encountered a different problem. They needed to determine whether an isolated skull belonged with a postcranial skeleton of a plesiosaur that had been removed from its wooden frame and disassembled. A photograph of a cast of the skeleton, in its original frame and with a skull, was known but the diagnostic features of the skull were not clear. The cast in the photograph, unfortunately, was lost or discarded at some point. But if another cast of the specimen could be found, the problem could be resolved [41].

Casts provide a three-dimensional view of a specimen, even for a specimen that is still in matrix, in contrast with a two-dimensional image in a photo or drawing. This results in more accurate measurements than can be obtained from a published figure [39]. As we have shown here, casts can be important for verifying the morphology depicted in figures from old publications, especially when the specimen is no longer available for study [40]. Moreover, old drawings and lithographs of specimens, which are often quite detailed, are an illustrator's interpretation of the specimen and thus can have inaccuracies, as we discussed above. In the Yale cast, for example, the rotation of proximal elements in the left forefin showed that the actual fin morphology differed from what was depicted on the published plate ([8] plate XV, fig. 1; figure 3*a,b*).

## 6. Conclusion

We have discovered two casts of the first ichthyosaur skeleton to have ever been formally recognized, named and figured [8], a practically complete specimen, probably found by Mary Anning in 1818. The original was almost certainly destroyed during an air raid in May 1941 during World War II.

Curiously, both existing casts of this historically important specimen are in collections outside of the UK, one in the USA and one in Germany. Records do not indicate when either of them was created, although it must have been between 1818, when the specimen was found, and 1930, when the cast was added to the collection of YPM. The Yale cast is typical of early nineteenth-century marine reptile specimens in being set into plaster and mounted in a wooden frame. It could be a very old cast, perhaps even dating back to when the specimen was in Colonel Birch's possession. The difference in quality of the casts indicates that they were not made at the same time. Furthermore, the Berlin cast has been painted to conform closely to the illustration. Certain elements were reinterpreted in the process, which led to some of the discrepancies discussed above. Although not in the best condition, the Yale cast is more accurate than either the painted Berlin cast or the original illustration.

The casts record morphological details that provide taxonomic information for this important fossil, although portions of the fins are not accurate in the Berlin cast and are not fully visible in the Yale cast. Notably, the shape of the left humerus, number of digits in the right forefin and the presence of a bifurcation of the anterior digit in the hindfin are reliable characters to identify the specimen as a species of *Ichthyosaurus* [2,22]. These same features can be seen in Home's illustration ([8], plate XV, fig. 1), but the casts verify their accuracy. However, the specimen cannot be assigned to one of the six valid species of *Ichthyosaurus* because diagnostic characters from the skull and postcranium are not preserved [18,42,43].

That both casts described herein were unidentified in their respective collections highlights the importance of specialists visiting and studying collections to provide expertise to curatorial staff, which unfortunately have been greatly reduced in numbers over the past decade or more. It also points to the potential significance of old casts in museum collections. We hope that this article might encourage visiting researchers and curators to look at their casts more closely in the hope of finding additional examples of the Home specimen.

## Institutional abbreviations

**MB.R.**, Museum für Naturkunde Berlin, Germany (Museum of Natural History); **YPM**, Yale Peabody Museum, New Haven, CT, USA.

## Data Availability

Supplementary material is available online [44].
